# Application of light microscopical and ultrastructural immunohistochemistry in the study of goblet cell carcinoid in the appendix

**DOI:** 10.1186/1477-7819-6-15

**Published:** 2008-02-06

**Authors:** Maya V Gulubova, Yovcho Yovchev, Tatyana Vlaykova, Philip Hadjipetkov, Diana K Prangova, Angel Popharitov

**Affiliations:** 1 Department of General and Clinical Pathology, Medical Faculty, Trakia University, Stara Zagora, 11 Armeiska Str., Stara Zagora, Bulgaria; 2 Department of General Surgery, Medical Faculty, Trakia University, Stara Zagora, Bulgaria; 3 Department of Chemistry and Biochemistry, Medical Faculty, Trakia University, Stara Zagora, Bulgaria

## Abstract

**Background:**

Goblet cell carcinoids appear less frequently in the appendix than do other carcinoids. In the presented work a case with a goblet cell carcinoid of the appendix is described.

**Methods:**

Routine histological and histochemical methods were employed, with a combination of histochemistry and immunohistochemistry on one section and light and electron microscopical immunohistochemisty on paraffin-embedded material, were applied to identify the type of the carcinoid and to reveal the fine structure of cell types in the tumour nests of the appendix.

**Results:**

During the biopsy of a patient who had undergone appendectomy, an infiltration with clusters of goblet cells in the submucosa of the appendix was found. After a second operation of right-sided hemicolectomy, similar clusters of goblet cells were detected in the muscle layers of the caecum. After 18 months the patient died from cirrhosis and had not developed metastases or any recurrence. Immunohistochemically the serotonin-, somatostatin-, chromogranin A- and synaptophysin-positive endocrine cells were basally attached to mucin-secreting cells. The combined staining revealed simultaneously present endocrine cells (chromogranin-A-positive) and mucin-secreting cells (PAS- or alcian blue-positive). The ultrastructural immunohistochemistry showed that chromogranin A-positive cells had discoid and pleomorphic granules and were located in tumour nests or as single cells in the appendiceal wall.

**Conclusion:**

The combined histochemical and immunohistochemical procedure and the ultrastructural immunohistochemistry on archival material could contribute in clarifying the diagnosis of goblet cell carcinoid.

## Background

In the last 30 years, histochemical, immunohistochemical and electron microscopic techniques were applied in the study of carcinoids of the appendix. With the aid of previously mentioned techniques an endocrine cell component has been detected in these tumours. In clinical aspect, published articles have predominantly addressed the diagnostic procedures, progression of, and therapy for the entity [1-4].

Goblet cell carcinoids appear in the appendix less frequently than other carcinoids (and constitute approximately 5% of all appendicle primary tumours) [1,3,5,6]. The goblet cell carcinoid is characterized histologically by goblet cells or signet ring-like cells arranged in clusters, separated by smooth muscle or stroma [2,3]. The endocrine cells are arranged basally in tumour glands [5]. Goblet cell carcinoids were considered more aggressive than classical carcinoids [2,3].

In order to determine the clinical behaviour for this tumour there existed several criteria such as low grade of differentiation, increased mitotic activity, invasion in the caecum, lymph nodes metastases and tumour size larger than 2 cm [7]. The right hemicolectomy was prevalent in a number of patients with goblet cell carcinoid [7,8]. In the last years an adjuvant chemotherapy was applied in the treatment of this type of carcinoid [9].

Almost all of the studies concerning precise diagnosis of goblet cell carcinoids, were histological and histochemical [1,6], or immunohistochemical [2,3,10]. The endocrine component of that carcinoid was shown to be positive for chromogranin A, serotonin, glucagon and pancreatic polypeptide [2,3,10]. The data about the ultrastructural studies were scarce [11]. We did not find an ultrastructural immunohistochemical study on this type of carcinoid published in English. Our report describes a combined histochemical and immunohistochemical technique and simultaneously presents the mucinous and the endocrine cell components of the goblet cell carcinoid on light microscopical paraffin sections. Ultrastructural immunohistochemistry on a paraffin-embedded specimen from goblet cell carcinoid was applied to reveal the fine structure of cell types in the tumour nests of the appendix.

## Methods

### Pathology

A 60 year old man diagnosed as having an acute perforative appendicitis and periappendicular abscesses, was treated with surgery. The pathological diagnosis was a goblet cell carcinoid of the appendix (WHO histological classification 8243/3), infiltrating the mesoappendix. The *macroscopic finding *consisted in a slightly tight, oval area in the submucosa of the appendix, located near the caecum and measuring about 0.3 cm in diameter. Concomitant liver cirrhosis (proven hostologically) was observed. Light microscopical finding was present in many groups of goblet cells, separated by fibrous stroma in the submucosa and the muscle layer of the appendix. Small pools of mucin were found between the cell nests. Some tumour nests had central lumens, mimicking normal crypts.

After four months the patient was treated with a second operation, right-sided hemicolectomy. The macroscopic appearance of the colon was almost normal. Only slight induration was observed in the submucosa of the caecum, at the place of the previous appendiceal resection. Histologically, the muscle layer and the submucosa of the caecum were diffusely infiltrated by goblet cells arranged in clusters and separated by fibrous stroma. In the wall of the caecum single tumour cells and nests infiltrated the myenteric plexus. Nuclear atypism and mitoses were visible.

After the right-sided hemicolectomy the patient was treated with six courses of 5 fluorouracil and leucovorin. In the 18 month period image analysis did not reveal metastases or recurrence. The patient was admitted to the hospital where he died from decompensated liver cirrhosis resulting in variceal oesophageal bleeding and with an autopsy confirming no recurrence of tumour.

### Methods

#### Routine histology

The sections were stained with hematoxyllin and eosin.

#### Histochemistry

Mucins in the lumen of tumour nests and in the goblet cells stained positively with PAS reaction and alcian blue.

### Light and electron microscopical immunohistochemistry

Earlier the floating section immunohistochemistry methodology was described [12]. The two procedures were carried out simultaneously and according to the method of De Vos *et al*. [13] on samples embedded in paraffin. In brief: paraffin sections 5 μm thick for light microscopical immunohistochemistry mounted on slides and 40 – 60 μm thick for electron microscope immunohistochemistry were prepared. They were dewaxed twice in xylene for 30 minutes at 56°C, followed by descending ethanol series. The sections were then soaked overnight in 10% sucrose solution at 4°C. The sections were also incubated in 1.2% hydrogen peroxide in methanol for 30 min, and rinsed in phosphate balanced solution (PBS), pH 7.4, for 15 min. The sections were then blocked for 30 min with normal mouse serum (DAKO). After incubating with the primary mouse (rabbit) anti-human antibodies overnight, the cryostat sections were washed in PBS and incubated with a secondary anti-mouse (rabbit) biotinylated antibody (DAKO) for 4 h, and subsequently with the streptavidin-HRP complex (DAKO) for 4 h, rinsed in PBS, and then in 0.05 M Tris-HCl buffer, pH 7.5, for 10 min. The reaction was made visible by using a mixture of 3 mg 3,3'-diaminobenzidine (DAB) (DAKO), in 15 ml 0.05 M Tris-HCl buffer, pH 7.5, and 36 μl 1% hydrogen peroxide for 10–20 min, and rinsed in PBS.

After dehydration the paraffin sections were mounted with entellan for light microscopy. For better visualization of the DAB reaction product the sections were not counterstained. Sections incubated with non-immune sera instead of the primary antibodies were used as negative controls.

The free floating sections (40–60 μm thick) were postfixed in PBS containing 2% osmium tetroxide for 30 min at 2°C, followed by a rinse in PBS. Finally, sections were dehydrated in graded concentrations of ethanol and propylene oxide, and flat-embedded with Durcupan, between celophane sheets. Ultrathin sections were cut from areas with immune reactive endocrine cells visible on cellophane preparations. For better visualization of the DAB reaction product they were not counterstained with uranyl acetate. Ultrathin sections were examined and photographed with an OPTON EM 109 electron microscope at 50 kV.

### A combined histochemical and immunohistochemical staining

After deparaffinization the 5 μm thick sections were stained first with PAS-reaction or with toluidine blue. Then, the preparations were not mounted with Kanada balsam. They were hydrated in PBS, pH 7.4 for 10 min. Endogenous peroxidase was quenched with 1.2% hydrogen peroxide in methanol for 30 min, and rinsed in PBS, pH 7.4, for 15 min. Then, the sections were incubated overnight with the rabbit anti-human chromogranin A, or with the mouse anti-human serotonin. After washing them in PBS, pH 7.4, incubation with a secondary anti- rabbit (mouse) biotinylated antibody (DAKO) for 4 h was done, and subsequently with the streptavidin-HRP complex (DAKO) for 4 h. They were rinsed in PBS, pH 7.4, and then in 0.05 M Tris-HCL buffer, pH 7.5, for 10 min. Finally the reaction was developed with DAB solution as was described above. The sections were mounted with entellan. The pink or blue colour of mucins (PAS or alciane blue) remained visible. Brown endocrine cells could be observed at the basement membrane, beneath goblet cells in the nests.

### Immunochemicals

The antibodies used were: rabbit anti-human chromogranin A (N1535), rabbit anti-human synaptophysin (N1566), mouse anti-human synaptophysin (U0037), rabbit anti-human somatostatin (N1551), and mouse anti-human serotonin (N1530), all obtained from DAKO A/S Denmark. The rabbit anti-human gastrin (PA019-5P), rabbit anti-human bombesin (PA062-5P), rabbit anti-human secretin (PA067-5P) and rabbit anti-human β-endorphin (PA063-5P) were obtained from BioGenex Laboratories, San Ramon, CA, USA. The detection system used was DAKO LSAB^®^2 System, HRP (K0675), and DAKO^®^DAB Chromogen tablets (S3000) (DAKO A/S Denmark).

## Results

### Histology

**Figure 1:**
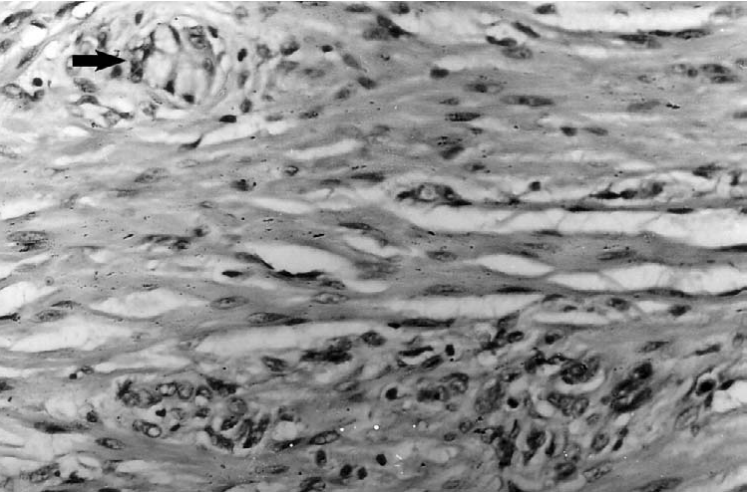
Clusters of goblet cells (**arrow**) infiltrated the muscle layer of the appendix. (Hematoxyllin and eosin). Magnification × 300.

The submucosa and the muscle layer of the appendix were diffusely infiltrated by goblet cells, arranged in clusters, and separated by fibrous stroma (Figure 1). Small pools of mucin were found between the cell nests. Tumour nests had central lumens, mimicking normal crypts. In the wall of the caecum single tumour cells and nests infiltrated the myenteric plexus, the muscle layer, and its submucosa. Nuclear atypism was visible.

### Light microscopic immunohistochemistry

**Figure 2:**
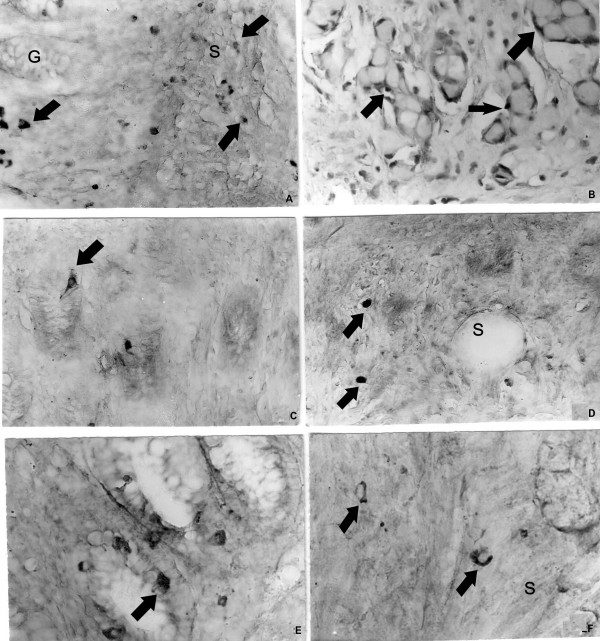
**a**. Chromogranin A-positive cells (**arrows**) in the normal appendiceal glands (**G**) and in submucosa (**S**), **b**. Chromogranin A-positive endocrine cells (**arrows**) delineate the tumour nests of goblet cells, **c**. Somatostatin-positive endocrine cells (arrows) in the normal appendiceal mucosa, **d**. Somatostatin-positive cells (**arrows**) in the appendiceal submucosa (**S**), **e**. Synaptophysin-positive endocrine cells (**arrow**) in the normal appendiceal mucosa, **f**. Synaptophysin-positive endocrine cells (**arrow**) in the appendiceal submucosa (**S**). Magnifications × a, b, c, d, e, f- × 300.

**Figure 3:**
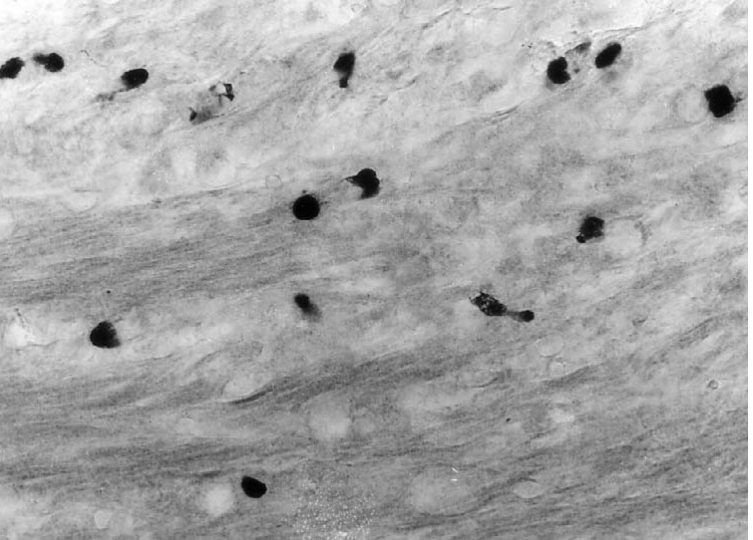
Serotonin-positive endocrine cells in the muscle layer of the caecum. Magnification × 300.

Dispersed endocrine cells or endocrine cells in nests containing 3–4 goblet cells were observed in the submucous and muscle layer of the appendix. The endocrine cells in appendiceal tumour nests were chromogranin A- (Figure 2a,b), somatostatin- (Figure 2c,d), synaptophysin- (Figure. 2e,f) and serotonin-positive. The endocrine cells, invading the wall of the caecum were all chromogranin A-, synaptophysin- and serotonin- (Figure 3) positive. The endocrine cells in the appendix and caecum tumour samples were bombesin-, endorphin-, gastrin- and secretin-negative.

### A combined histochemical and immunohistochemical staining

**Figure 4:**
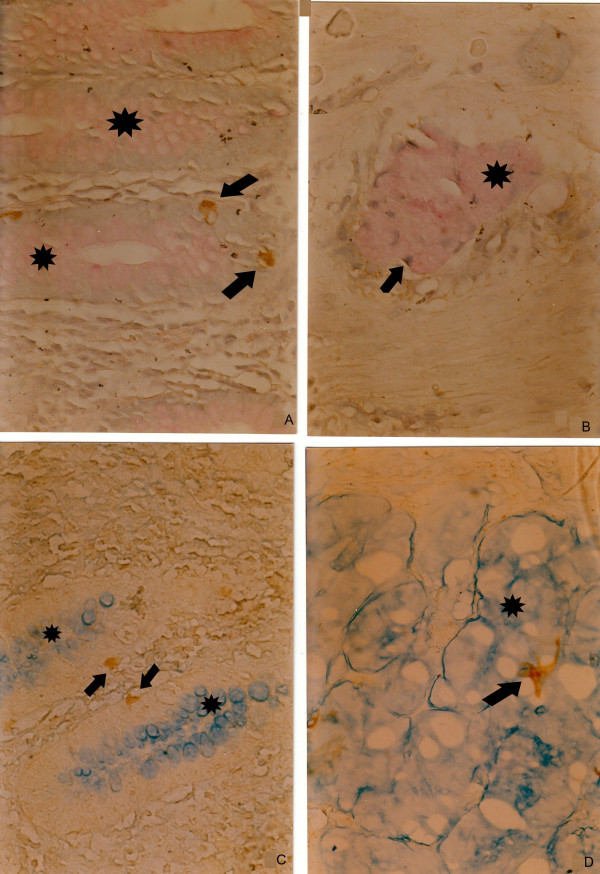
**a**. PAS-positive mucous cells (**pink, star**), and brown chromogranin A-positive endocrine cells (**arrow**) in the normal appendiceal mucosa, **b**. PAS-positive mucin-secreting cells (**pink, star**), surrounded by brown chromogranin A-positive endocrine cells (**arrow**) in a tumour gland in the submucosa, **c**. Alciane blue-positive mucous cells (**blue, star**) and brown chromogranin A-positive endocrine cells (**arrow**) in the normal appendiceal mucosa, **d**. Alciane blue-positive mucin-secreting cells (**blue, star**), and brown chromogranin A-positive endocrine cells (**arrow**) in a tumour gland. (A combined histochemical and immunohistochemical staining). Magnifications a, b, c d × 300.

PAS-positive mucous cells were surrounded by brown chromogranin A-positive endocrine cells (Figure 4a,b). Brown serotonin-positive cells were attached to alcian blue-positive mucous cells (Figure 4c,d).

### Ultrastructural immunohistochemistry

**Figure 5:**
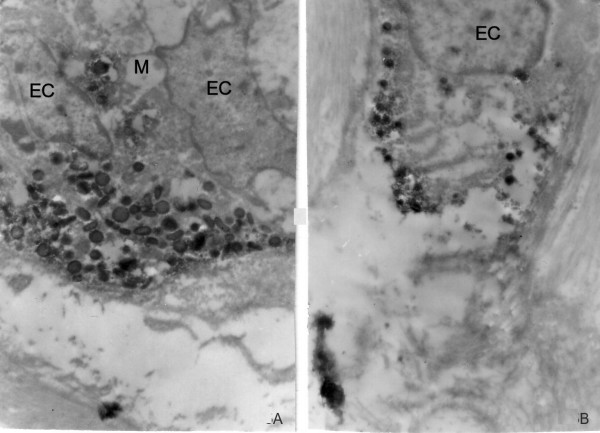
**a**. Two chromogranin A-positive endocrine cells (**EC**) in a tumour gland with discoid and pleomorphic granules, located basally to mucus-secreting cells (**M**), **b**. Single chromogranin A-positive endocrine cell (**EC**), infiltrating the submucosa. Magnifications a × 7000, b × 7 000.

Tumour nests resembling the normal crypts could be seen in the submucosa of the appendix. The mucous cells showed slight nuclear atypia. The endocrine cells were gathered in groups of 2 or 3 and were located basally to the mucous cells (Figure 5a). Their granules contained chromogranin A reaction product and were from the ovoid or discoid EC_2 _type. Single chromogranin A-positive endocrine cells, likely from D type with small electron-dense ovoid granules were found in the stroma of the submucosa (Figure 5b).

## Discussion

In the appendix goblet cell carcinoids appear less frequently than conventional carcinoids [3]. A review of appendiceal tumours set their incidence at only 5% of occurring appendiceal primary tumours [14]. A small number of goblet cell carcinoids have been already described: 30 cases [1]; 13 cases [10]; 10 cases [6]; 33 cases [2]. The existence of this entity is well documented [15] and many immunohistochemical and some ultrastructural studies have been reported [2,3,10,11].

The goblet cell carcinoid shares histological features with adenocarcinoma (abundant mucin production) and with conventional intestinal carcinoids (endocrine cells). In our case the neoplastic elements were located in the submucosa, as are conventional carcinoids [1]. In contradiction to adenocarcinoma the mucosa was free of malignant changes.

Our patient first had symptoms of acute appendicitis, as was found in most cases described in existing literature [3]. Our 60 year old patient conformed to the median age of patients with such tumours, as literature reports as being over 54.1 years old. The other carcinoid types in the appendix occurred in patients of approximately 40 years old [1].

In our case the surgical resection of the right colon was based on histological data of submucosal infiltration of the appendiceal wall, upon the finding of mitoses and nuclear atypism [16]. The histological appearance of the tumour in our case is identical with previously described, like tumours (goblet cells arranged in clusters and separated by connective tissue stroma) [2,3,6]. The presence of mucin secretion in the goblet cells was confirmed by stained PAS-reaction and with toluidine blue, as was aforementioned [3,6].

The immunohistochemical analyses showed endocrine cells, immunoreactive for serotonin, somatostatin and for the pan endocrine markers chromogranin A and synaptophysin, which were dispersed among the groups of goblet cells infiltrating the submucosa and muscle layer of appendix. Similar clusters of goblet cells and immunoreative endocrine cells were also found in the wall of the ceacum.

Goblet cell carcinoids display no clear distinguishing immunohistochemical pattern [2,3]. In our case the appendiceal tumour was diffusely positive for chromogranin A, serotonin and synaptophysin. Somatostatin immunoreactivity was found in scattered cells. It is known that tubular carcinoid is stained weakly for chromogranin A [5], while goblet cell carcinoids are stained more intensively for the same substance [3]. Irregular serotonin and somatostatin immunoreactivity in these tumours was reported earlier [3,10]. To our knowledge synaptophysin immunoreactivity was not investigated in goblet cell carcinoids. Synaptophysin like chromogranin is a universal marker of neuroendocrine cells [17]. We observed a diffuse synaptophysin immune reaction in the endocrine cells of the goblet cell carcinoid. Synaptophysin immunoreactivity was visualized also ultrastructurally in cells with granules of the EC_2 _type.

Ultrastructural examination revealed tumour nests with well-differentiated mucus-producing cells delineated by 2–3 endocrine cells with basement membrane location. The nests were in the submucosa. The endocrine cells were attached to goblet cells or were dispersed as single cells in the appendiceal wall. Electron microscopic examination of our case failed to reveal existence of cells that contain both mucin and secretary granules within their cytoplasm. Therefore we agree with the hypothesis of the dual entodermal and neuroendocrine origin of goblet cell carcinoid [1]. The ultrastructural investigation showed that in morphology the endocrine cells were from the EC_2 _type with discoid and pleomorphic granules [18] or from the D type with small electron-dense ovoid granules [19]. We found that chromogranin A marked the endocrine cells from these two types. The ultrastructural immunohistochemistry carried out in the current study was performed on archival materials from paraffin blocks. In this respect, we suggest this method as a suitable tool to study the hormonal nature of goblet cell carcinoids, the location of hormones and the phenotype of endocrine cells.

Another useful method for simultaneous revealing of mucin secretion and endocrine cell component of the tumour on archival paraffin blocks is the combined PAS/alcian blue/chromogranin A staining, applied in our current work. Earlier, Hosaka et al. [20] had used a similar combination of immunohistochemical (with anti-chromogranin A) and histochemical (PAS/alcian blue) method for a simultaneous detection of endocrine cells and mucin-secreting cells, to present a case with an early-stage colon adenocarcinoma with neuroendocrine differentiation. These authors first performed the immunohistochemical procedure and then counterstained sections with the PAS/alcian blue solution. We transposed the procedures. We first made PAS or alcian blue staining and then the immunohistochemistry. We demonstrated that the immunohistochemical procedure could be performed on previously PAS or alciane blue stained sections, allowing use of immunohistochemistry on archival sections, where paraffin blocks were lost or cut out. The use of combined special histochemical staining methods and immune reactions showed that the mucin-containing goblet cells were sharply delineated from the endocrine cells.

## Conclusion

Based on our results we find out that apart from the described serotonin-, somatostatin- and chromogranin A-positive endocrine cells, the goblet cell carcinoid contains also synaptophysin-positive endocrine cells. The ultrastructural immunohistochemistry showed mainly cells from the EC_2 _or D type. The combined histochemical and immunohistochemical procedure delves a greater possibility for revealing the dual nature of goblet cell carcinoide.

## Competing interests

The author(s) declare that they have no competing interests.

## Authors' contributions

MVG has made substantial contributions to conception, design, practical laboratory work, acquisition, analysis and interpretation of data, and drafting of the manuscript. MVG has given final approval of the version to be published. AP has contributions to acquisition of clinical data, to interpretation of data, follow up of the patient and to drafting of the manuscript. YY, PH and DKP have contributed to acquisition of clinical and pathological data, follow up of the patient and interpretation of data. TV has made contributions to practical laboratory work, technical preparation of the manuscript and its critical revision.

All authors have read and approved the final manuscript.
